# Orthodontic Retreatment of an Adult Bimaxillary Protrusion Patient With Lingual Appliances and Premolar Extractions: A Case Report

**DOI:** 10.7759/cureus.65983

**Published:** 2024-08-02

**Authors:** Viet Anh Nguyen

**Affiliations:** 1 Faculty of Dentistry, Phenikaa University, Hanoi, VNM

**Keywords:** esthetic appliances, passive self-ligating, premolar extraction, lingual appliances, case report

## Abstract

Orthodontic relapse and the demand for improved esthetics often necessitate retreatment in adult patients. This case report highlights the successful management of an adult female patient with bimaxillary protrusion, previously treated with a non-extraction approach. Treatment included lingual passive self-ligating appliances, premolar extractions, and mini-screws to reinforce anchorage. The patient's facial profile and dental esthetics were significantly improved after 20 months of treatment, achieving a stable occlusion and reduced bimaxillary protrusion. This case demonstrates that lingual appliances can be an effective and esthetic treatment option for complex orthodontic retreatment in adult patients. Careful biomechanical planning and attention to specific challenges, including torque control and bowing effect, are essential for successful outcomes.

## Introduction

The demand for orthodontic retreatment has increased recently in adult patients despite comprehensive orthodontic treatment received during their childhood or adolescence [1]. The objectives of retreatment may be to address relapses of dental crowding or spacing due to poor retention compliance. Additionally, retreatment may be required to improve dental and facial esthetics, which are inadequately addressed in prior orthodontic treatment, possibly due to an improper treatment plan [2].

Adult patients seeking orthodontic retreatment may have higher expectations regarding treatment time, results, and appliance esthetics due to their prior orthodontic experience [3,4]. This heightened awareness can make them more discerning when choosing a treatment plan and appliance options. Esthetic appliances such as lingual brackets and clear aligners might be preferred to traditional labial appliances.

This case report presents the orthodontic retreatment of an adult patient with mouth protrusion that was not alleviated in the previous non-extraction treatment. The retreatment included premolar extractions, lingual appliances, and mini-screws.

## Case presentation

Diagnosis and etiology

A 32-year-old female patient presented with a chief complaint of mouth protrusion. She had undergone orthodontic treatment with fixed labial appliances five years ago using a non-extraction strategy. However, she remained dissatisfied with the outcome as the bimaxillary protrusion remained significant. Her dental history revealed a history of dens evaginatus on the mandibular left second premolar, which had been worn out during chewing.

**Figure 1:**
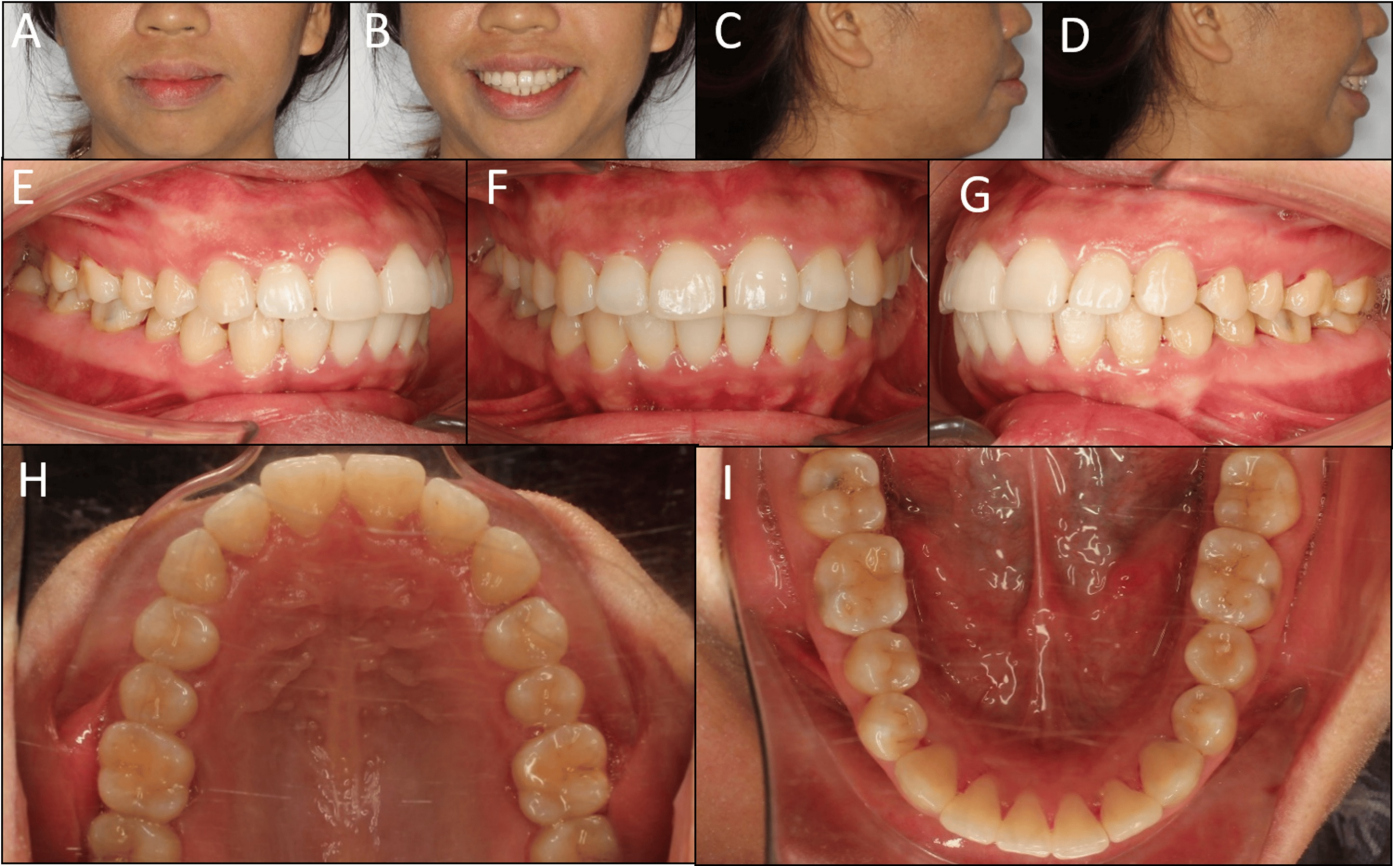
Pre-treatment extraoral and intraoral photographs. (A) Frontal, (B) frontal smiling, (C) lateral, (D) lateral smiling, (E) right occlusion, (F) anterior occlusion, (G) left occlusion, (H) upper arch, (I) lower arch.

On the extraoral examination, the patient had a convex profile with protruded lips and mentalis strain on lip closure. The vertical proportion of the face was balanced, with a left-deviated chin (Figure 1). Her smile arc was flat, with normal buccal corridors, suggesting that arch expansion was not necessary [5]. Mini-esthetic analyses, including tooth size and shape, gingival contour, contact, and embrasure, revealed no abnormality. No signs of temporomandibular disorders were detected.

On the intraoral examination, the patient had class I canine and molar relationships on both sides with normal overjet and overbite. There was approximately a 0.4 mm space between the upper central incisors and a 0.5 mm space between the upper left canine and first premolar. The lower second molars were lingually inclined with low positions of lingual cusps. The lower left second premolar showed a worn extra cusp and did not respond to the pulp test.

On the lateral cephalometric evaluation, the patient had a class II skeletal relationship (point A-nasion-point B: 7.0°) with a protruded maxilla (A to N perpendicular: 7.1 mm), a retruded mandible (sella-nasion-point B: 75.2°), and a normodivergent facial pattern (Frankfort mandibular angle: 26.9°). The upper incisors had a normal inclination (upper incisor/sella-nasion: 103.1°) while the lower incisors were proclined (incisor mandibular plane angle: 112.5°, interincisal angle: 104.7°) (Table 1).

**Table 1:** Lateral cephalometric measurements. SNA, sella-nasion-point A; SNB, sella-nasion-point B; ANB, point A-nasion-point B; FMA, Frankfort mandibular angle; SN-MP, sella-nasion to mandibular plane; SN, sella-nasion; NA, nasion-point A; MP, mandibular plane; NB, nasion-point B.

Measurements	Pre-treatment	Post-treatment	Norm
Skeletal			
SNA (°)	82.2	82.0	81.1 ± 3.7
SNB (°)	75.2	75.4	79.2 ± 3.8
ANB (°)	7.0	6.6	2.5 ± 1.8
FMA (°)	26.9	26.1	25.0 ± 4.0
A to N perpendicular (mm)	7.1	5.7	0.4 ± 2.3
Dental			
Upper incisor/SN (°)	103.1	95.4	105.3 ± 6.6
Upper incisor/NA (°)	18.9	14.4	22.0 ± 5.0
Upper incisor/NA (mm)	5.9	1.1	4.0 ± 3.0
Lower incisor/MP (°)	112.5	99.3	90.0 ± 3.5
Lower incisor/NB (°)	49.4	34.3	25.0 ± 5.0
Lower incisor/NB (mm)	19.4	8.7	4.0 ± 2.0
Interincisal angle (°)	104.7	129.7	128.0 ± 5.3
Upper incisal display (mm)	3.3	1.5	2.5 ± 1.5
Overjet (mm)	2.1	2.0	2.0 ± 2.0
Overbite (mm)	0.7	1.0	2.0 ± 2.0
Soft tissue			
E-line/Upper lip (mm)	2.4	0.4	0.0 ± 2.0
E-line/Lower lip (mm)	7.7	2.0	0.0 ± 2.0

**Figure 2:**
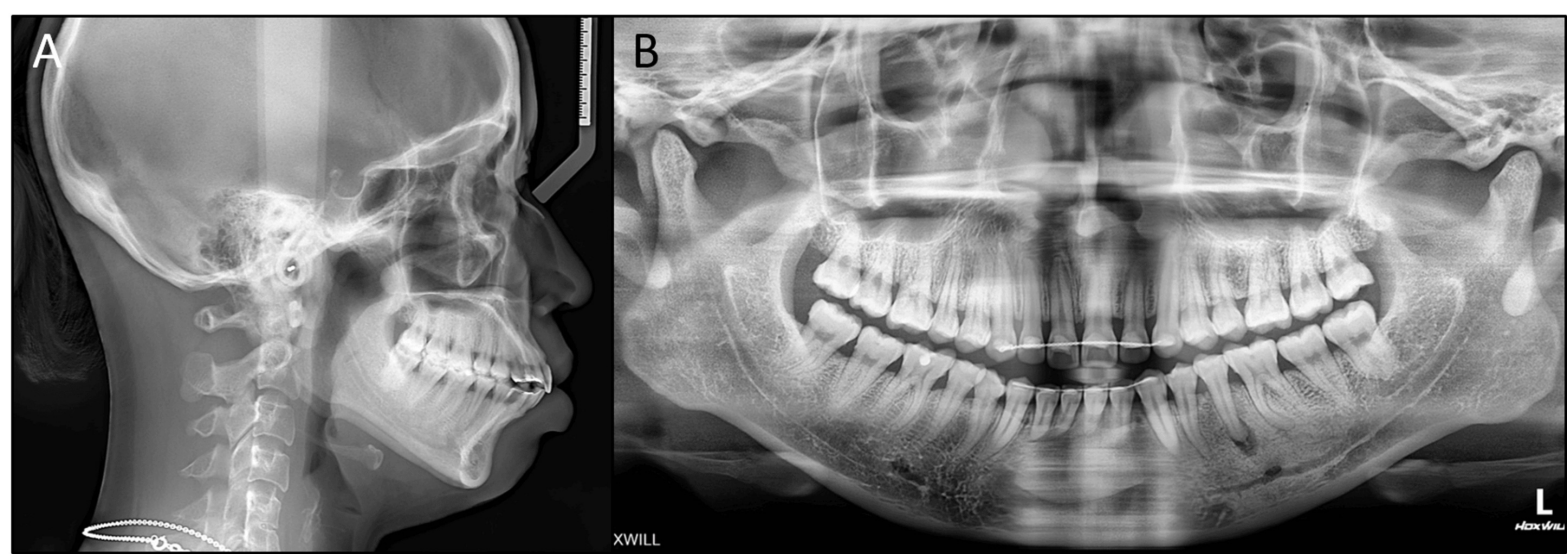
Pre-treatment radiographs. (A) Lateral cephalometric radiograph, (B) panoramic radiograph.

The panoramic radiograph showed the presence of all teeth including the third molars, a periapical radiolucency on the lower left second premolar, and the lingual fixed retainers on both arches (Figure 2). Additionally, condylar degeneration resorption was apparent despite the absence of clinical signs and symptoms.

Treatment plan

The treatment objectives were (1) to retract the upper and lower incisors to reduce lip protrusion, (2) to close all spaces in both arches, (3) to correct the lingually inclined lower second molars, and (4) to maintain the class I dental relationship, normal overbite, and overjet.

Considering the amount of dentoalveolar and lip protrusion, the normal inclination of the upper incisors, the proclined lower incisors, and the skeletal class II relationship, the optimal treatment plan was an orthodontic-orthognathic combination. The orthodontic decompensation would include premolar extractions in both arches followed by maxillary anterior segmental osteotomy and mandibular bilateral sagittal split osteotomy to address the protruded maxilla. The second treatment alternative was a non-surgical strategy with extractions of the upper first premolars, lower left first premolar, and hopeless lower left second premolar to retract the anterior teeth in both arches, compensating for the dentoalveolar protrusion. The second treatment alternative was selected as the patient denied surgical intervention. Lingual esthetic appliances were chosen as requested by the patient.

Treatment progress

Treatment was initiated by bonding all teeth with 0.018” x 0.025” lingual passive self-ligating brackets (Linpass SL, ADB, Korea) except for the four premolars to be extracted. Double vacuum-formed thermoplastic indirect bonding trays were used [6]. Mushroom lingual archwires were used in the initial leveling and alignment stage with the sequence of 0.014”, 0.016”, and 0.016” x 0.022” nickel-titanium wires.

**Figure 3:**
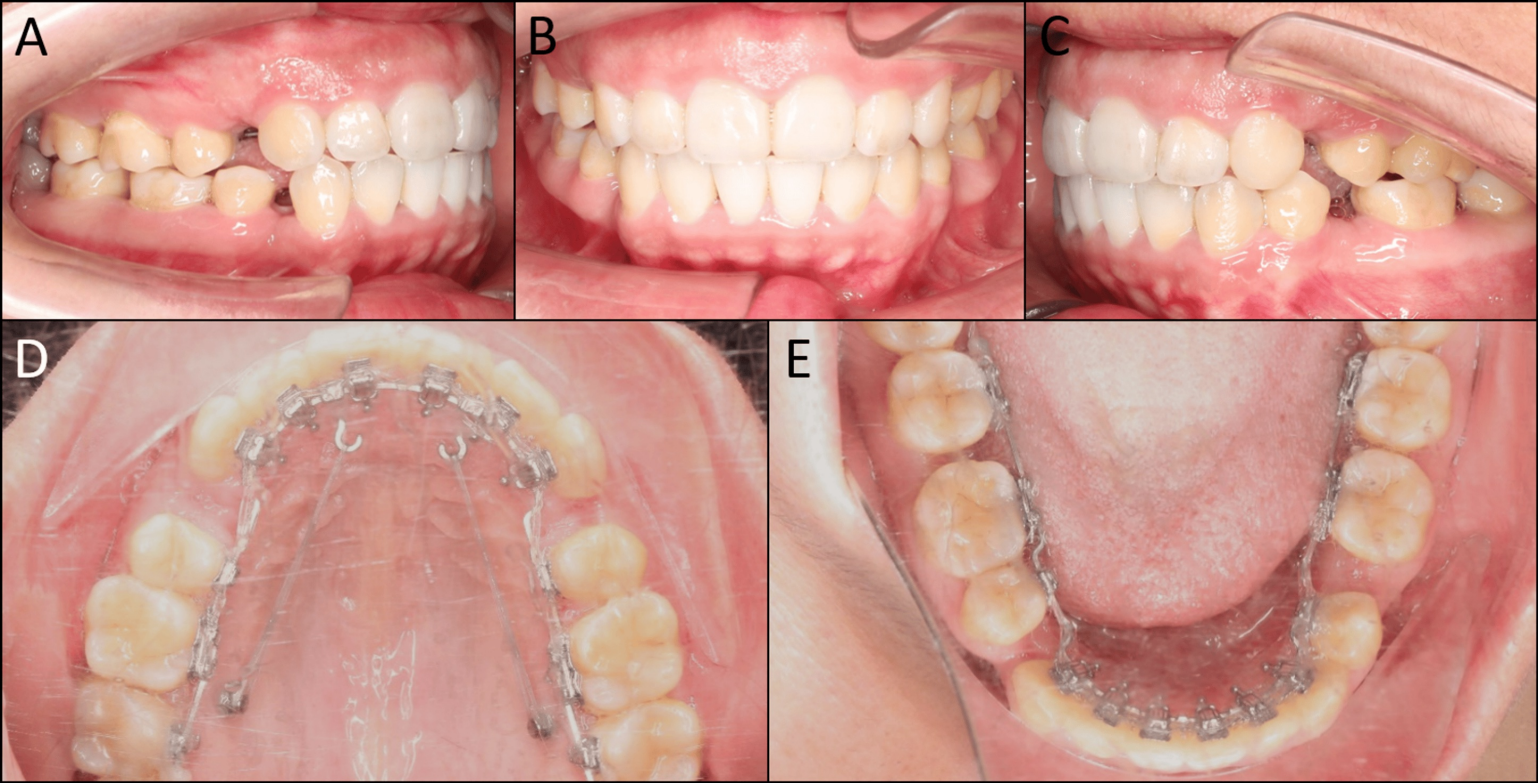
Space closure. (A) Right occlusion, (B) anterior occlusion, (C) left occlusion, (D) upper arch, (E) lower arch.

After four months of treatment, the upper first premolars, lower right first premolar, and hopeless lower right second premolar were extracted. Stiff 0.016” x 0.022” stainless steel archwires with incorporated reverse curves of Spee were engaged to start the space closure stage. The upper archwire was pre-torqued with 15 degrees for torque control of the upper incisors. Power chains were used to retract the upper and lower incisors. Mini-screws and crimpable hooks were used in the upper arch to reinforce the posterior anchorage due to the class II tendency (Figure 3).

After six months of initial space closure, the offset bends interfered with the first molar brackets, hindering continued space closure. Therefore, the old archwires were replaced with new ones with offset bends placed right posteriorly to the second premolar brackets. Despite the incorporated curves of Spee in the archwires, a mild lateral open bite developed due to the vertical bowing effect. Hence, vertical elastics were applied to the lingual bracket to close the lateral open bite.

A 20-degree pre-torqued 0.017” x 0.025” stainless steel archwire was engaged in the upper arch to fully express the torque of the upper incisors in the last three months of the 13-month space closure stage. The finishing stages took another three months and the total active treatment time was 20 months. After lingual appliance removal, fixed retainers were boned in both arches along with clear vacuum-formed retainers for nighttime wear.

Treatment results

**Figure 4:**
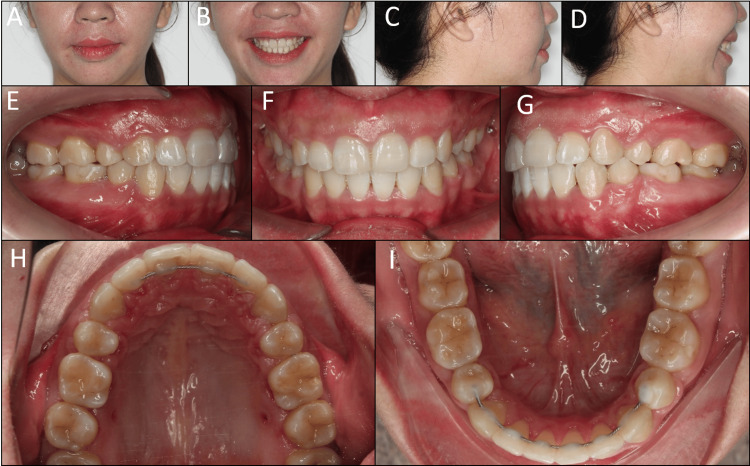
Post-treatment extraoral and intraoral photographs. (A) Frontal, (B) frontal smiling, (C) lateral, (D) lateral smiling, (E) right occlusion, (F) anterior occlusion, (G) left occlusion, (H) upper arch, (I) lower arch.

Post-treatment extraoral and intraoral photographs show satisfied results with balanced smile and facial esthetics and functional occlusion (Figure 4). The lip protrusion was significantly reduced without mentalis strain on lip closure. The smile arc became more consonant. The class I canine and molar relationships were well-maintained with a normal overjet, overbite, and solid cuspal interdigitation. All extraction spaces were completely closed.

**Figure 5:**
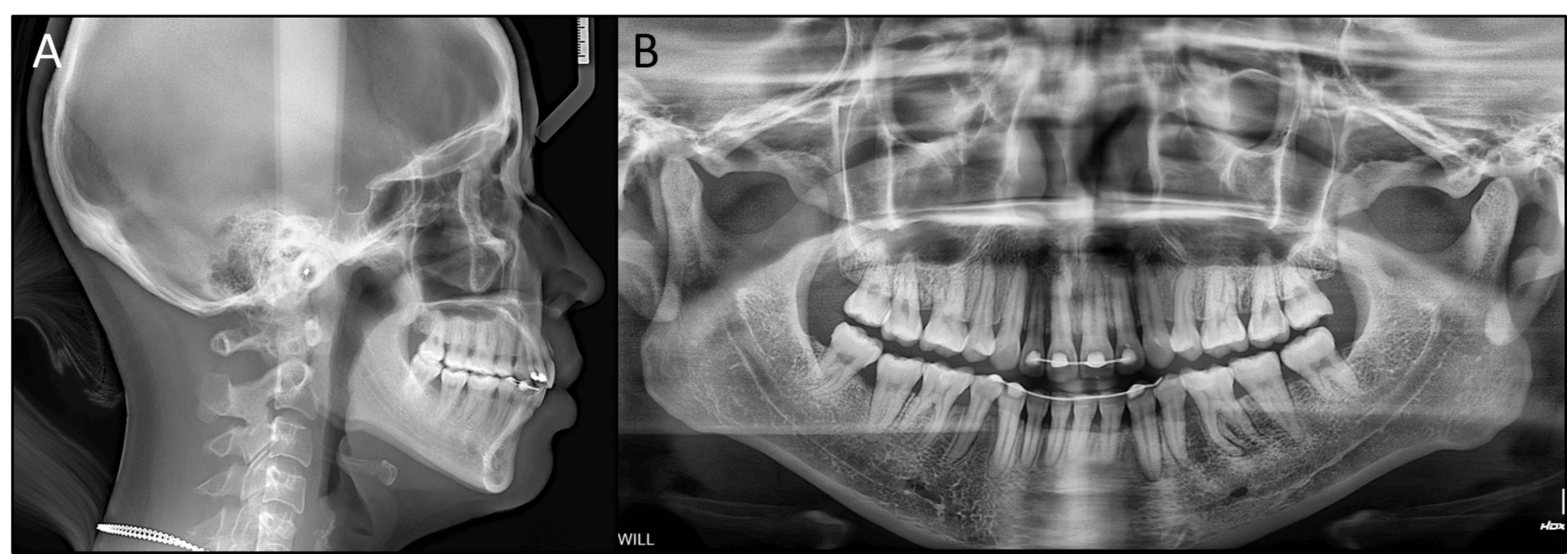
Post-treatment radiographs. (A) Lateral cephalometric radiograph, (B) panoramic radiograph.

The lateral cephalometric analysis showed a slight improvement in the skeletal class II relationship (point A-nasion-point B: 6.6°) and maxillary protrusion (A to N perpendicular: 5.7°). The lower incisor proclination was significantly reduced (incisor mandibular plane angle: 99.3°). Despite lingual tipping occurring on the upper incisors, the torque control of these teeth was acceptable as their roots approximated the lingual cortical bone (Figure 5). The panoramic radiograph showed acceptable root parallelism without signs of root resorption.

**Figure 6:**
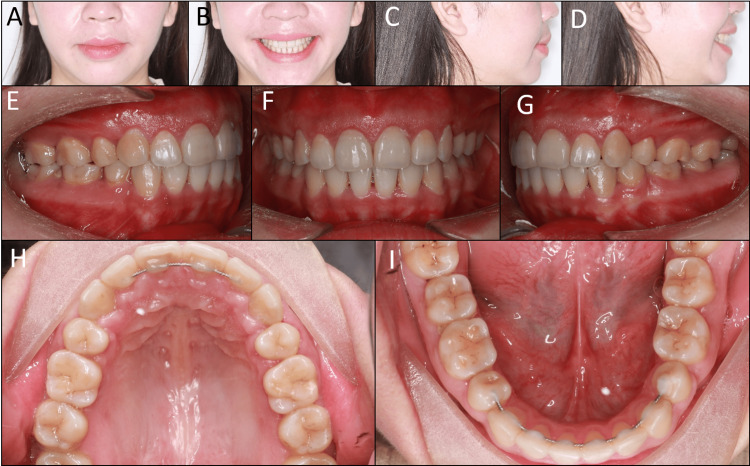
One-year post-retention extraoral and intraoral photographs. (A) Frontal, (B) frontal smiling, (C) lateral, (D) lateral smiling, (E) right occlusion, (F) anterior occlusion, (G) left occlusion, (H) upper arch, (I) lower arch.

On the six-month follow-up, a gingivectomy was performed on the upper arch as requested by the patient to correct her short teeth. A digitally planned guide for precise gingival removal was designed an manufactured [7]. The one-year post-retention extraoral and intraoral photographs show the treatment outcomes to be stable without relapse tendency (Figure 6).

## Discussion

In this case report, the patient sought a second orthodontic treatment due to dissatisfaction with prior non-extraction treatment. Despite achieving class I occlusion and well-aligned dental arches, the patient's dentoalveolar protrusion persisted, worsening their lateral profile and lip incompetence, and negatively impacting facial and smile esthetics.

In our practice, lingual appliances are generally the appliances of choice of adult patients seeking orthodontic retreatment due to their invisibility. This is especially true in cases requiring premolar extractions as a higher success rate could be expected with fixed appliances compared to clear aligners [8,9]. Within Eastern Asian patients seeking retreatment, dentoalveolar protrusion is a common cause as it has a high occurrence risk in patients treated with a non-extraction strategy. Eastern Asian female patients are generally not satisfied with convex profiles and proclined teeth [10]. Mild to moderate crowding cases could be managed with a non-extraction strategy with molar distalization and interproximal reduction [11,12]. However, decision-making may become challenging in borderline cases, possibly leading to an inappropriate non-extraction plan.

**Figure 7:**
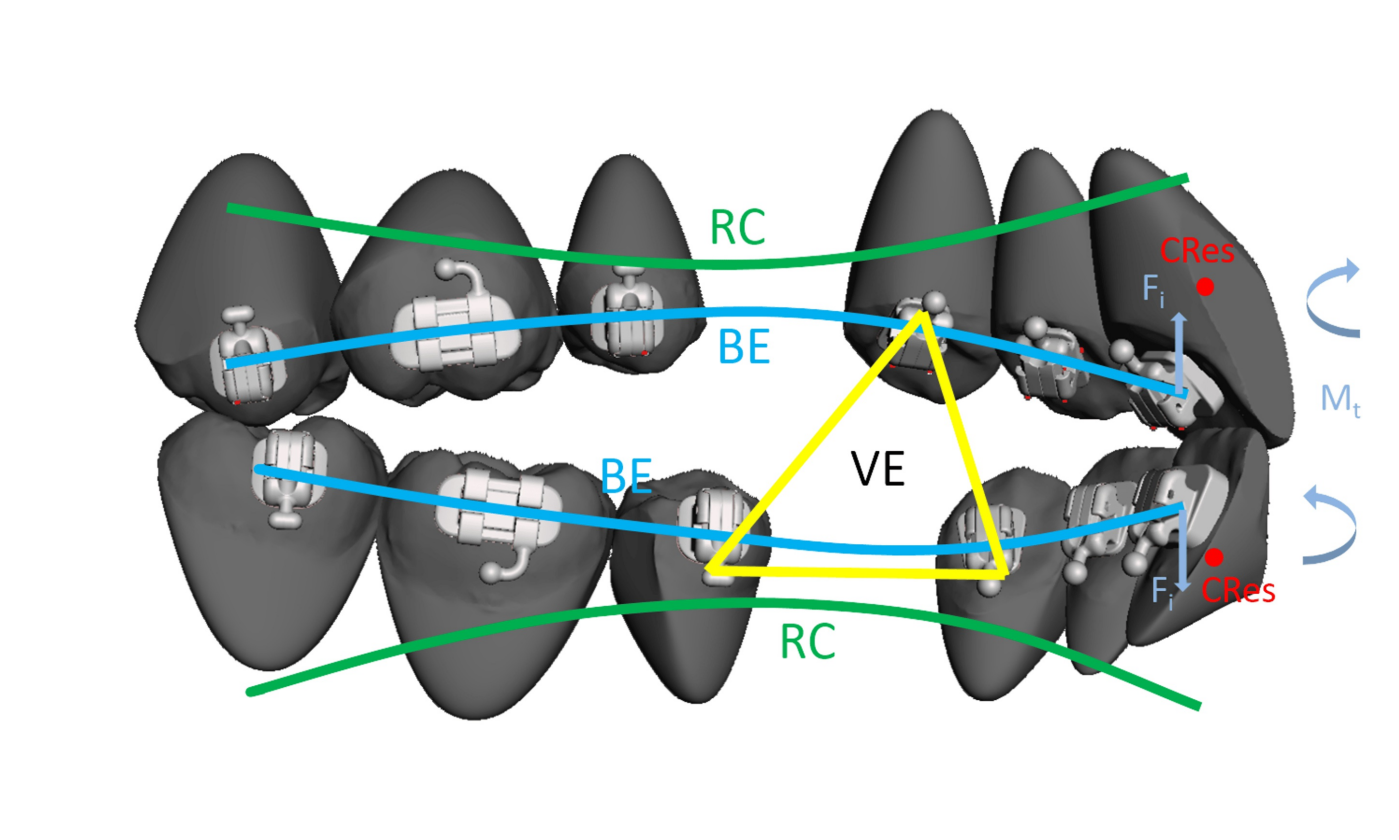
Biomechanics of space closure with lingual appliances. The bowing effect (BE) tends to create a lateral open bite. Flat shapes of archwires apply intrusion forces (Fi) to incisors passing lingually to incisors’ centers of resistance (CRes), resulting in lingual tipping moments (Mt). Reverse curves of Spee (RC) incorporated within archwires and vertical elastics (VE) can neutralize the bowing effect and avoid lingual tipping of incisors.

On the other hand, lingual appliances may pose some difficulties in premolar extraction cases compared to labial appliances. The most prominent challenge is the tendency for lingual tipping of the incisors during retraction [13]. The reason for this phenomenon is that the intrusion force of the archwire applied to the incisor lingual brackets passes lingually to the center of resistance of the incisors, in contrast to the labial brackets [14,15]. The intrusion force is generated by the archwire, as the incisors tend to extrude due to the vertical bowing effect (Figure 7). In this case report, the torque control of the lower incisors was straightforward due to their pre-treatment proclination. However, the torque control of the upper incisors posed more challenges because of their normal pre-treatment inclination. Nevertheless, the use of pre-torqued archwires with incorporated curves of Spee in combination with power hooks and vertical elastics led to a satisfactory result, with the upper incisor roots well placed in the cancellous bone approximately the lingual cortical plate.

Another challenge of lingual appliances in managing premolar extraction cases is the interference of offset bends of the mushroom archwires with the first molar brackets during space closure, necessitating archwire replacement. To minimize this, the offset bends should be placed as close to the second premolar brackets as possible. Recently, lingual straight archwires have been introduced to eliminate offset bends and potentially offer higher stiffness to reduce the bowing effect [16-18]. However, these straight archwires may require increased tooth-bracket distance, potentially leading to thicker adhesive and a higher risk of bracket failure, along with potential patient discomfort due to the increased profile. Therefore, the classical mushroom archwire technique was used in this patient.

## Conclusions

An adult patient with bimaxillary protrusion, previously treated with a non-extraction approach, was successfully retreated using lingual appliances, premolar extractions, and mini-screws. Significant improvements in both facial and dental esthetics were achieved. This case report demonstrates the potential of lingual appliances for effective and esthetic retreatment of compromised treated orthodontic cases in adult patients. Careful control of anchorage, incisor torque, and bowing effect is essential for achieving successful outcomes in extraction cases. Pre-torqued archwires with incorporated reverse curves of Spee, power hooks, and vertical elastics may be necessary in cases of challenging torque control.
